# Differences in infection and prevention of HIV and other sexually transmitted infections among older adults in Columbus, Ohio

**DOI:** 10.1371/journal.pone.0282702

**Published:** 2023-03-06

**Authors:** Ethan Morgan, Christina Dyar, Brian A. Feinstein

**Affiliations:** 1 College of Nursing, The Ohio State University, Columbus, OH, United States of America; 2 Infectious Disease Institute, The Ohio State University, Columbus, OH, United States of America; 3 Department of Psychology, Rosalind Franklin University of Medicine and Science, North Chicago, IL, United States of America; Fairfield University Marion Peckham Egan School Of Nursing & Health Studies, UNITED STATES

## Abstract

**Introduction:**

In the United States, rates of sexually transmitted infections (STIs) have increased year after year for the past six consecutive years. Even so, the majority of research has focused on younger populations with little work examining infections and prevention methods among older adults.

**Methods:**

Data come from the Columbus Health Aging Project (N = 794). This study was designed to assess several domains of health among adults aged 50 years and older in Columbus, Ohio with a particular focus on addressing disparities based on sexual and gender identity. Multivariable logistic regression models were used to examine the association between sociodemographic factors and risk of STI acquisition, HIV diagnosis, and several common prevention methods, adjusting for known confounders.

**Results:**

Key results suggest that cisgender women, intersex individuals, and transgender women are less likely to use condoms relative to cisgender men. Meanwhile, white individuals were least likely to use condoms while bisexual individuals were most likely. Transgender women and those living with family/roommates were most likely to use PrEP/PEP relative to cisgender men and those living with a spouse or partner. Cisgender women, compared to cisgender men, were most likely to report not using any prevention method.

**Conclusion:**

This study highlights the need for better research among older adults in order to ascertain how interventions may be targeted to specific populations. Future research should aim to educate individuals differently based on their specific needs rather than treating older adults as a homogenous population or ignoring their sexually active nature entirely.

## Introduction

In the United States (U.S.), rates of sexually transmitted infections (STIs; e.g., syphilis, gonorrhea, chlamydia) have increased year after year for the past six consecutive years [1, 2]. These increases have resulted in an incidence of over 26 million acquired STIs translating to nearly 1 in 5 adults in the U.S. being diagnosed [3]. More specifically, compared to 2017, STI rates in 2018 have risen: chlamydia increased 2.7%, gonorrhea 5.0%, syphilis 3.3%, and congenital syphilis 39.7% [1]. Although STIs have broadly increased in recent years, HIV has simultaneously experienced a decline over the same period. Rates of HIV infection have decreased from a relatively stable incidence of 50,000–58,000 cases per year during the period from 1991 to 2007 down to 34,800 cases in 2019 [4]. This decrease is likely attributable in large part to the approval and uptake of HIV preventative medications such as pre-exposure prophylaxis (PrEP) [5]. Across HIV and other STIs, however, disparities continue to persist based on several factors such as sex, gender identity, race/ethnicity, and sexual minority status (e.g., gay, lesbian, bisexual, non-binary, and other non-heterosexual identities). Importantly, this area of research remains vastly understudied among older adults likely due to a persistent, inaccurate assumption that they are sexually inactive [6, 7].

The most recent data available from the Centers for Disease Control and Prevention (CDC) highlight extant disparities across all STIs and age groups [2]. Recent trends in primary and secondary syphilis cases highlight this point. In 2019, overall cases of primary and secondary syphilis increased by 11.2% relative to 2018 [2]. Disparities by race and ethnicity continue to persist with the lowest rates among people identifying as Asian (4.6 per 100,000) followed by those identifying as white (6.6 per 100,000), Hispanic (13.7 per 100,000), American Indian and Alaska Native (21.2 per 100,000), Native Hawaiians and Other Pacific Islander (23.0 per 100,000), and Black (31.0 per 100,000) [2]. Long term trends between men and women (2015–2019) continue to demonstrate stark disparities with men (13.8 to 20.1 per 100,000) experiencing much higher rates of primary and secondary syphilis relative to women (1.4 to 3.9 cases per 100,000 population) [2]. And although risk of incident infection remains highest among young adults aged 25–29, rates of infection among adults 55 or older have increased drastically in recent years by approximately 103%, yet little research has focused on this population [2]. Across the board, similar trends in disparities can be seen among other STIs including both gonorrhea and chlamydia. Even in light of this, however, the majority of population-based research continues to focus on younger populations of MSM and transgender women [8–12] to the detriment of older adults, particularly in terms of types of HIV/STI prevention methods used.

HIV diagnoses meanwhile have experienced an overall steady decline in recent years, although Black individuals continue to make up the largest proportion of new HIV diagnoses in the U.S. today at 41% [4]. Meanwhile, the only racial or ethnic group to experience an increase over the same period is Hispanic people who have steadily risen over time to make up 29% of incident HIV infections [2]. Both women and heterosexual people, typically low risk groups, have experienced a similar rise in the proportion making up new HIV diagnoses. In 2019, women accounted for 18% of new diagnoses (up from 8% at the onset of the outbreak) and heterosexual people accounted for 22% of new diagnoses (from 2% at the onset of the outbreak) [4]. When examining trends by age and gender during the period 2015 through 2019, only women aged 55 and older who saw a rise at 7% while men 55 and older experienced a stable trend over the same period [13, 14].

Research on sexual health among older adults has remained limited in large part due to persistent misconceptions that older adults experience low sexual activity. In fact, past studies have demonstrated that large proportions of both older women and men remain active well into their elderly years [6, 7]. One potential reason for this stereotype may be a reluctance among providers to collect sexual activity history from among older adults [15], a practice (or lack thereof) which may have the unintended consequence of driving the erroneous conclusion that older adults are not sexually active. This may also result in limiting providers’ ability to communicate new or updated methods of preventing acquisition of HIV or STIs and may also result in reductions in research among this population.

While national reports, such as those compiled yearly by the CDC, aid in shedding light on incidence rates of HIV and other STIs among older adults, more in-depth research is needed at the community level to begin to design targeted prevention aimed at disrupting health disparities. And although the total number of cases remain lower, understudied populations such as middle-age and older adults (aged ≥50) have experienced larger percentage increases in STIs than their younger counterparts yet make up one of the smallest foci of current research in this area, even less so among mid-sized U.S. cities such as Columbus. To begin to address this gap, we recruited a large, pilot cohort of adults aged ≥50 in order to examine disparities in HIV and STI testing, diagnoses, and prevention methods across sex, gender identity, race and ethnicity, and sexual minority status.

## Methods

### Study population

Data come from the cross-sectional pilot survey, the Columbus Healthy Aging Project (CHAP). CHAP was designed to assess several domains of health (e.g., HIV/STI, substance use) and potential risk factors (e.g., stress, stigma) among adults aged 50 years and older in Columbus, Ohio. Recruitment occurred throughout the Columbus metropolitan area exclusively via Facebook and Instagram. As this study has a particular focus on assessing disparities between sexual minorities and heterosexuals two ads were run: one for the general population and a second targeted towards sexual minorities. Inclusion criteria included: 1) age ≥50 years; 2) residence in Columbus, Ohio or surrounding suburbs; 3) access to a computer or smartphone to complete the online survey assessment; and 4) a working email address. We also aimed to recruit equal numbers of sexual minority and heterosexual participants while maintaining a racially and ethnically diverse sample that was reflective of Columbus’s own distribution. This was done by pre-defining categories of participants that matched the racial and ethnic make-up of Columbus, stratified by gender and sexual minority status (e.g. non-Hispanic Black sexual minority women, etc). Once recruitment was completed for each category, no other participants were considered eligible. All participants provided electronic informed consent and were compensated $20 in the form of an Amazon gift card for their time. All study protocols and procedures were approved by The Ohio State University’s Institutional Review Board (2020B0394).

### Demographic measures

Demographic information was self-reported by participants and included age, sex, gender identity, race, ethnicity, and sexual identity. Age was self-reported and operationalized as a continuous variable. Sex was reported as female, male, or intersex and coded as such. Gender identity was similarly self-reported as cisgender, transgender, or a different identity and coded as such. Race and ethnicity were coded based on participant self-identification as American Indian/Alaska Native, Asian, Black, Hispanic/Latinx, Native Hawaiian/Other Pacific Islander, white, Multiracial, or a different race. The variable used in this analysis was operationalized as Black, white, Hispanic/Latinx, or a different racial identity. Any individual self-identifying as Hispanic/Latinx was categorized as such regardless of race, consistent with CDC surveillance reports [1, 2]. Sexual identity was operationalized as gay or lesbian, bisexual, heterosexual, or a different identity. Participants were also asked which situation best describes their current living situation, the variable operationalized as living alone, living with family members or roommates (non-romantic partners), living with a partner or spouse (romantic partner), or living in a long-term care facility. Living with family members or roommates were separately assessed and combined into a single category.

### Sexual health measures

Participants self-reported whether they were ever tested for HIV or any other STIs in their lifetime as well as in the past six months, and each variable was operationalized dichotomously (Note: CDC recommendations for HIV testing are once per year among those aged 13–64 [16]). Those who reported being tested for either HIV or STIs in the past six months were subsequently prompted to report whether the test was positive or negative, and the variable was dichotomized as HIV-negative or HIV-positive and positive or negative for any STI. Participants were also asked to self-report the use of any HIV/STI prevention methods in the past six months, including: condoms, withdrawal/pull-out, pre-exposure or post-exposure prophylaxis (PrEP/PEP) use, spermicidal foam/jelly/cream/film/suppository, diaphragm, female condom/vaginal pouches, no methods used, or a different method used. The survey question was asked such that participants were able to select all that applied while each method was separately operationalized as a dichotomous variable. Each of these were also compared to their direct counterparts as the reference category (e.g., condom use compared to no condom use, PrEP/PEP use compared to no PrEP/PEP use).

### Statistical analyses

Univariate participant characteristics were described using means, standard deviations, and proportions, as appropriate. Multivariable logistic regression models were then utilized to assess health disparities adjusting for demographic characteristics and other risk factors. Specifically, we assessed five models examining differences in: 1) any positive STI test; 2) HIV status; 3) use of condoms; 4) use of PrEP/PEP; and 5) no HIV/STI prevention methods used. The final three analyses were chosen as they were the most common prevention methods reported by participants. Models were selected based on a mixture of statistical testing (those variables significant at the p < 0.05 level), confounding assessment, and *a priori* knowledge regarding risk factors for both HIV and STI transmission. Statistical significance was established at alpha = 0.05. All analyses were performed in StataBE 17.0.

## Results

Table 1 presents demographic characteristics of the sample (N = 794). Mean age was 58.5 years (standard deviation [SD] = 6.3). Overall, 371 (49.0%) participants were assigned female at birth, 375 (49.5%) were assigned male at birth, and 11 (1.5%) were intersex. The majority of the sample identified as cisgender (n = 690, 86.9%) while 52 (6.6%) identified as transgender and 52 (6.6%) as a different gender identity. Regarding race and ethnicity, 19 (2.4%) identified as American Indian or Alaska Native, 14 (1.8%) as Asian, 297 (37.4%) as Black, 49 (6.2%) as Hispanic or Latinx, none as Native Hawaiian or other Pacific Islander, 411 (51.8%) as white, and 4 (0.5%) as Multiracial or a different race. There were 394 (49.6%) participants who identified as gay or lesbian, 52 (6.6%) as bisexual, 317 (39.9%) as heterosexual, and 31 (3.9%) as a different sexual identity.

**Table 1:** Demographic attributes of the Columbus Healthy Aging Project, Columbus, OH (N = 794).

	N (%)	Mean (SD)
**Age**	–	58.5 (6.3)
**Sex**		
Female	371 (49.0)	–
Male	375 (49.5)	–
Intersex	11 (1.5)	–
**Gender Identity**		
Cisgender	690 (86.9)	–
Transgender	52 (6.6)	–
Different Identity	52 (6.6)	–
**Race and Ethnicity**		
American Indian/Alaska Native	19 (2.4)	–
Asian	14 (1.8)	–
Black	297 (37.4)	–
Hispanic/Latinx	49 (6.2)	–
Native Hawaiian/Other Pacific Islander	0 (0.0	–
White	411 (51.8)	–
Multiracial or different race	4 (0.5)	–
**Sexual Identity**		
Gay or Lesbian	394 (49.6)	–
Bisexual	52 (6.6)	–
Heterosexual	317 (39.9)	–
Different Identity	31 (3.9)	–
**Sexually Transmitted Infections**		
STI test, ever	414 (53.0)	
STI test, past 6 months	192 (46.4)	
Any positive STI	30 (15.6)	
**HIV**		
HIV test, ever	438 (56.5)	
HIV test, past 6 months	165 (37.9)	
Number of tests, past 6 months		2.4 (2.1)
HIV-positive status^1^	32 (7.3)	
**Prevention Methods, past 6 months** ^**2**^		
Condoms	408 (51.8)	
Withdrawal/Pull-out	84 (10.7)	
PrEP/PEP	118 (15.0)	
Spermicidal foam/jelly/etc	57 (7.2)	
Diaphragm	13 (1.7)	
Female condom or vaginal pouch	77 (9.8)	
None	219 (27.8)	
Other	37 (4.7)	
**Living Situation**		
With Spouse/Partner	326 (40.6)	
Alone	169 (21.1)	
With Family/Roommate	269 (33.5)	
Long-Term Care	14 (1.7)	
Other	25 (3.1)	
**Health Insurance Status**		
None	175 (22.3)	
Have Insurance	610 (77.7)	

^1^Percent of those who have had at least one test in their lifetime

^2^Participants were able to select multiple options

Regarding sexual health history, 192 (46.4%) participants reported having a test for any STI in the past six months with 30 (15.6%) of those testing positive. 165 (37.9%) reported being tested for HIV in the past six months with 32 (7.3%) participants ever having been diagnosed with HIV. Condoms were the most commonly reported prevention method used (408, 51.8%) followed by those who reported using no preventative method (219, 27.8%).

Table 2 presents a series of logistic regressions examining the associations between demographic variables and: 1) any positive STI test compared to none, 2) HIV-positive status relative to HIV-negative status, 3) use of condoms to prevent HIV/STIs compared to no condom use, 4) use of PrEP/PEP compared to using neither, and 5) lack of any preventive method used relative to the use of at least one prevention method. We observed no significant demographic differences (i.e., age, sex and gender, race and ethnicity, or sexual identity) in self-reporting positive for any STIs. Regarding HIV status, older age was significantly associated with HIV-positive status (adjusted odds ratio [aOR] = 1.07; 95% confidence interval [CI]: 1.00, 1.14). Compared to gay/lesbian participants, heterosexuals were significantly less likely to report an HIV-positive status (aOR = 0.06; 95% CI: 0.01, 0.27), no bisexuals reported a positive diagnosis for HIV.

**Table 2:** Multivariable logistic regressions assessing self-reported HIV and STI test results and select prevention methods.

	STIs & HIV		Prevention		
	Any Positive STI	HIV-Positive Status	Condom Use	PrEP/PEP Use	No Method Used
**Age**	1.08 (0.99, 1.18)	1.07* (1.00, 1.14)	0.91*** (0.88, 0.94)	0.99 (0.95, 1.02)	1.09*** (1.06, 1.13)
**Sex and Gender**					
Cisgender Men	Ref	Ref	Ref	Ref	Ref
Cisgender Women	1.81 (0.73, 4.49)	0.70 (0.29, 1.71)	0.31*** (0.21, 0.45)	0.88 (0.53, 1.43)	3.00*** (1.97, 4.59)
Intersex	–^^	–^^	0.20* (0.04, 0.96)	–^^	3.66 (0.73, 18.21)
Transgender Women	–^^	–^^	0.17*** (0.06, 0.45)	4.77** (1.85, 12.34)	0.18 (0.02, 1.41)
Transgender Men	–^^	5.21 (0.33, 81.43)	0.99 (0.37, 2.63)	0.69 (0.17, 2.80)	0.86 (0.28, 2.69)
Different Identity	0.86 (0.16, 4.69)	0.20 (0.02, 1.58)	0.51 (0.26, 1.00)	1.43 (0.66, 3.12)	0.09 (0.01, 0.72)
**Race/Ethnicity**					
White	Ref	Ref	Ref	Ref	Ref
Black	1.67 (0.67, 4.14)	0.60 (0.25, 1.42)	3.48*** (2.40, 5.06)	0.57* (0.35, 0.93)	0.28*** (0.18, 0.44)
Hispanic/Latinx	0.90 (0.21, 3.92)	0.48 (0.10, 2.39)	3.97*** (1.97, 8.01)	0.24 (0.05, 1.04)	0.05*** (0.01, 0.22)
Different Race/Ethnicity	–^^	–^^	2.31* (1.07, 4.97)	1.70 (0.67, 4.32)	0.30* (0.12, 0.76)
**Sexual Identity**					
Gay/Lesbian	Ref	Ref	Ref	Ref	Ref
Bisexual	1.24 (0.16, 9.68)	–^^	2.17* (1.01, 4.68)	0.73 (0.27, 2.00)	0.96 (0.39, 2.40)
Heterosexual	0.40 (0.15, 1.06)	0.06*** (0.01, 0.27)	1.03 (0.71, 1.50)	0.49** (0.30, 0.82)	3.09*** (2.02, 4.71)
Different Identity	–^^	–^^	1.11 (0.44, 2.79)	0.80 (0.26, 2.44)	2.14 (0.76, 6.00)
**Living Situation**					
With Spouse/Partner	–	–	Ref	Ref	Ref
Alone	–	–	1.31 (0.84, 2.05)	1.07 (0.56, 2.02)	0.77 (0.45, 1.29)
With Family/Roommate	–	–	1.56* (1.05, 2.32)	2.72*** (1.63, 4.55)	0.57* (0.35, 0.92)
Long-Term Care	–	–	1.76 (0.50, 6.24)	8.88** (2.08, 38.00)	0.59 (0.13, 2.60)
Other	–	–	0.83 (0.26, 2.64)	0.81 (0.10, 6.60)	0.57 (0.13, 2.40)
**Health Insurance Status**					
None	–	–	–	Ref	–
Have Insurance	–	–	–	2.79** (1.47, 5.33)	–

^Gender identity omitted due to no transgender individuals being HIV-diagnosed

^^Cells empty, no individuals

We then examined the three primary methods of HIV/STI prevention, condom use, use of PrEP or PEP, and lack of any method used (Table 2). Older age was inversely, significantly associated with condom use (aOR = 0.91; 95% CI: 0.88, 0.94). Meanwhile, compared to cisgender men, cisgender women (aOR = 0.31; 95% CI: 0.21, 0.45); intersex participants (aOR = 0.20; 95% CI: 0.04, 0.96), and transgender women (aOR = 0.17; 95% CI: 0.06,0.45), were each less likely to report condom use. Relative to white participants, black (aOR = 3.48; 95% CI: 2.40, 5.06), Hispanic/Latinx (aOR = 3.97; 95% CI: 1.97, 8.01), and those identifying as a different race/ethnicity (aOR = 2.31; 95% CI: 1.07, 4.97) were each significantly more likely to report condom use. Similarly, bisexuals (aOR = 2.17; 95% CI: 1.01, 4.68) and those living with family members or a roommate (aOR = 1.56; 95% CI: 1.05, 2.32) were each significantly more likely to report condom use compared to gay or lesbian individuals and those living with a spouse or partner.

Next we examined the use of PrEP/PEP (Table 2). Transgender women, compared to cisgender men, were significantly more likely to report the use of PrEP/PEP (aOR = 4.77; 95% CI: 1.85, 12.34). Meanwhile, black participants (aOR = 0.57; 95% CI: 0.35, 0.93) and heterosexual participants (aOR = 0.49; 95% CI: 0.30, 0.82) were each significantly less likely to report use of PrEP/PEP compared to white participants and those identifying as gay or lesbian. Compared to those living with a spouse or partner, those living with family or a roommate (aOR = 2.72; 95% CI: 1.63, 4.55) and those living in long-term care facilities (aOR = 8.88; 95% CI: 2.08, 38.00) were both significantly more likely to report PrEP/PEP use.

The final model in this series examined the lack of any use of prevention methods during sexual encounters. We observed a significant association between older age and lack of prevention use (aOR = 1.09; 95% CI: 1.06, 1.13). Cisgender women (aOR = 3.00; 95% CI: 1.97, 4.59) and heterosexuals (aOR = 3.09; 95% CI: 2.02, 4.71) were both significantly more likely to report lack of preventative use relative to cisgender men and gay or lesbian individuals. Compared to white participants, those reporting as black (aOR = 0.28; 95% CI: 0.18, 0.44), Hispanic/Latinx (aOR = 0.05; 95% CI: 0.01, 0.22), or as multiracial or a different racial/ethnic identity (aOR = 0.30; 95% CI: 0.12, 0.76) were each significantly less likely to report using no HIV/STI prevention method.

## Discussion

This study examined disparities in HIV/STI testing, diagnosis, and use of prevention methods among a diverse sample of middle-aged and older adults in Columbus, OH. We observed several key findings including a lack of any significant difference in testing positive for STIs across all demographic categories in adjusted analyses. As expected, heterosexual participants were significantly less likely to report testing positive for HIV. Significantly less condom use was observed among cisgender women, intersex individuals, and transgender women relative to cisgender men while racial and ethnic minority participants and bisexual participants reported significantly greater condom use relative to their counterparts. Conversely, transgender women were more likely to report PrEP/PEP use while black individuals and heterosexuals was significantly less likely to report use of PrEP/PEP. Finally, older age was associated with lower likelihood of condom use and greater likelihood of using no other method of prevention.

Past research has demonstrated that misconceptions surrounding low sexual activity among older adults are incorrect. Large proportions of both older women and men remain active well into at least their eighties [6, 7] even though older women are less likely than their male counterparts to be report being sexually active [6]. Prior research assessing STIs among older adults has found that older women are significantly more likely than younger women to be diagnosed with STIs such as trichomonas while both older women and men are more likely to be diagnosed with late or unknown stage syphilis relative to their younger counterparts [7]. Meanwhile, adults over the age of 50 make up 17% of new diagnoses in the U.S. with nearly three-quarters of these among men [17]. However, none of this past work differentiates sex assigned at birth from current gender identity, shortcoming addressed by our research presented here. Interestingly, when examining sex assigned at birth among our sample of older adults, we little to know report of positive HIV or STI test. Among those reporting a positive test, particularly transgender men, we observed no significant difference relative to cisgender men. At a minimum, however, we did observe that cisgender women, transgender women, and intersex participants were less likely to use condoms compared to cisgender men. Only transgender women, however, were significantly more likely to report use of PrEP/PEP, a finding which may hint at a potential reduction in disparities among this population. Future research should focus more specifically on older transgender populations as neither community-based research nor CDC data provide detailed estimates on this potentially high-risk population.

Past research examining racial and ethnic differences among older adults are more mixed. One past study among a sample of older Black Americans noted that 40% were unaware whether condoms could prevent HIV or STIs and 98% viewed themselves as being at low-to-no risk of infection [18]. Another study noted an increase in the use of condoms among older Black participants reporting multiple sexual partners [19], suggesting knowledge of how to prevent HIV and STIs. Yet a third study conducted primarily among a sample of older white adults found broadly low knowledge regarding transmission and acquisition risk of HIV and STIs [20]. While we did not specifically address knowledge or self-perceived risk of HIV/STIs in this study, we did assess prevention methods used by our sample of older adults. In this, we found that both black and Hispanic/Latinx participants were significantly more likely to report use of condoms and less likely to report using no prevention methods in general. The only caveat to these results is that black participants were also less likely to reported PrEP or PEP use. These results suggest that, relative to past research, general knowledge may be high regarding STI transmission but that access to or knowledge about PrEP/PEP may need to be improved among racial and ethnic minority populations to reduce disparities in HIV infection. Other possibilities for the observed differences may be found in infrequent discussions of sexual health with one’s provider, a lack of equitable access to healthcare, or more simply a dearth of sexual education among older adults [6, 21–23]. Future work in this area should utilize extant research to develop targeted interventions aimed at improving education and knowledge among this population.

Older adults continue to face not only high risk of HIV/STI transmission but also face stigma and discrimination at the intersection of age and sexual minority status [24]. Poor awareness of one’s risk of HIV/STIs and lack of communication with medical professionals may impact overall use of preventative methods relative to younger populations [15]. Our own work here demonstrates this with bisexual individuals in our sample reporting greater condom use relative to gay and lesbian individuals while heterosexuals reported a greater likelihood of using no prevention methods at all. These findings suggest a few possibilities. First, they may be attributable to inconsistent messaging regarding sexual health among older adult populations, a finding which again may be as a result of poor communication with one’s provider, particularly among sexual minority populations.^11^ And second, it may be that intervention strategies have been treated homogenously and have lacked any nuance to the needs of specific populations (e.g., targeted to older adults versus younger adults, older sexual minorities versus older heterosexuals). Future research should be focused on developing age- and population-specific interventions to reduce disparities between high and low risk populations.

Our study should be considered in light of its limitations. First, these data are cross-sectional and thus limited in not being able to draw causal conclusions. Second, these data are self-reported thus subject to reporting biases (e.g., social desirability). Next, this is a sample limited in geography and may only reflect the local population rather than the entire population of older adults in the U.S. Our sample of intersex individuals was also very limited thus the findings regarding this population should be interpreted carefully, future research should aim to recruit a larger sample of this vastly understudied group of individuals. Third, despite our efforts to recruit a gender diverse sample of older adults, we were limited in our analyses by having no transgender individuals self-reporting testing positive for HIV or STIs. Last, we did not assess simultaneous use of prevention methods thus are unable to draw any conclusions regarding overlap of prevention methods. Future work should aim to focus more specifically on older transgender adults to better understand transmission among this population and appropriately tailor intervention efforts.

Even considering these limitations, we observed several key findings. First, as past research has noted, we continue to see disparities in HIV and STIs across sex, gender identity, race/ethnicity, and sexual identity, particularly in terms of type of prevention method used (or lack thereof). Although risk of HIV/STI transmission remains greatest among sexual minority individuals and individuals who use substances, the rising incidence of HIV/STIs [2] among previously low-risk populations such as older adults and heterosexuals highlights a growing need to better understand new diagnoses of each among this population. Second, this study highlights the need for better research among older adults to ascertain how interventions may be targeted to specific populations. Future research should aim to educate individuals differently based on their specific needs rather than treating older adults as a homogenous population, or worse, ignoring their sexually active nature entirely.
